# Survey Results and Recommendations from Japanese Stakeholders for Good Clinical Practice Renovation

**DOI:** 10.1007/s43441-021-00350-4

**Published:** 2021-11-17

**Authors:** Kenichi Nakamura, Hitoshi Ozawa, Taro Shibata, Nobuko Ushirozawa, Tomomi Hata, Natsuko Okita, Nozomu Fuse, Norihiro Sato, Koji Ikeda, Hideki Hanaoka, Tatsuya Maruyama, Michihiko Wada, Shinobu Shimizu, Hiroi Kasai, Yoichi Yamamoto, Jun Sakurai, Koji Todaka, Shimon Tashiro, Haruko Yamamoto

**Affiliations:** 1 Clinical Research Support Office, National Cancer Center Hospital, Tokyo, Japan; 2 Department of International Clinical Development, National Cancer Center Hospital, 5-1-1 Tsukiji, Chuo-ku, Tokyo, 104-0045 Japan; 3 Center for Research Administration and Support, National Cancer Center, Tokyo, Japan; 4 Clinical Research Support Office, National Cancer Center Hospital East, Chiba, Japan; 5 Clinical Research and Medical Innovation Center, Hokkaido University Hospital, Hokkaido, Japan; 6 Clinical Research, Innovation and Education Center, Tohoku University Hospital, Miyagi, Japan; 7 Clinical Research Centre, Chiba University Hospital, Chiba, Japan; 8 Clinical Research Support Center, The Tokyo University of Tokyo Hospital, Tokyo, Japan; 9 Clinical & Translational Research Center, Keio University Hospital, Tokyo, Japan; 10 Department of Advanced Medicine, Nagoya University Hospital, Aichi, Japan; 11 Institute for Advancement of Clinical and Translational Science, Kyoto University Hospital, Kyoto, Japan; 12 Department of Medical Innovation, Academic Clinical Research Center, Osaka University Hospital, Osaka, Japan; 13 Center for Innovative Clinical Medicine, Okayama University Hospital, Okayama, Japan; 14 Center for Clinical and Translational Research, Kyushu University Hospital, Fukuoka, Japan; 15 Department of Sociology, Graduate School of Arts and Letters, Tohoku University, Miyagi, Japan; 16 Department of Data Science, National Cerebral and Cardiovascular Center, Osaka, Japan; 17 Pharmaceuticals and Medical Devices Agency (PMDA), Tokyo, Japan

**Keywords:** International Council for Harmonisation of Technical Requirements for Pharmaceuticals for Human Use, Good Clinical Practice, Clinical trial regulation, Real-world data, Remote monitoring

## Abstract

**Background:**

The International Council for Harmonisation of Technical Requirements for Pharmaceuticals for Human Use (ICH) is undertaking a major revision of ICH E6 Good Clinical Practice (GCP) decided to involve external stakeholders in ICH-GCP renovation. Activities such as surveys and public conferences have taken place in the United States, European Union, and Japan. For stakeholder engagement in Japan, a designated research group conducted a survey of academic stakeholders.

**Methods:**

A total of 105 academic stakeholders from 18 institutions responded to the survey. The research group developed recommendations reflecting the survey results and the opinions from patients and the public.

**Results:**

The survey showed the top four principles needing renovation were (i) informed consent (Chapter 2.9, 12.4% of respondents believed it needed renovation), (ii) systems for quality assurance (Chapter 2.13, 9.5%), (iii) information on an investigational product (Chapter 2.4, 5.7%), and (iv) procedures on clinical trial information (Chapter 2.10, 5.7%). The top three sections identified as needing renovation were: (i) informed consent (Chapter 4.8, 27.6%), (ii) monitoring (Chapter 5.18, 22.9%), and (iii) composition, functions, and operations of the ethics committee (Chapter 3.2, 14.3%). Recommendations included clarification of ICH-GCP’s scope, proportionality in various aspects of clinical trials, diversity and liquidity of ethics committee members, modernization of informed consent procedures, variations in monitoring, and regulatory grade when using real-world data.

**Conclusion:**

The recommendations from Japanese investigators and patients have been submitted to the ICH E6 Expert Working Group, which will strengthen the robustness of the GCP renovation.

## Introduction

The E6 Good Clinical Practice (GCP) guidelines issued by the International Council for Harmonisation of Technical Requirements for Pharmaceuticals for Human Use (ICH) are widely accepted as a global standard for ensuring the protection of human subjects and reliability in clinical trials of medicinal products [1]. As a founding member of ICH, Japan has the second largest drug market in the world [2] and has been deeply involved in the revision of all ICH guidelines. ICH-GCP has been adopted in Japan as the Ministerial Ordinance on Standards for the Conduct of Clinical Trials on Medicinal Products (Japanese GCP). It is regarded as an extremely important rule for conducting industry-sponsored and investigator-initiated clinical trials, where the trial data are intended to be submitted to regulatory authorities for the new drug application.

The original ICH-GCP was enacted in 1996. It had not been fully amended for approximately 25 years, although an integrated addendum on risk-based monitoring and electronic document management was incorporated as Revision 2 (R2) in 2016. However, changes in the environment surrounding drug development and clinical trials have generated urgent demand for a major ICH-GCP revision. A paper entitled “ICH Reflection on ‘GCP Renovation’: Modernization of ICH E8 and Subsequent Renovation of ICH E6” was published in 2017 [3]. Subsequently, ICH-E6 (R3) was formally adopted as a new topic at the ICH Amsterdam Assembly in June 2019. The Management Committee approved a concept paper [4] and a business plan [5] at the November 2019 ICH meeting in Singapore. At that meeting, an expert working group (EWG) for ICH-GCP revision was established.

The ICH is an organization whose members include regulatory authorities and pharmaceutical industries, but its guidelines have a significant impact on other stakeholders such as academic researchers and patients. In the usual ICH guideline development process, input from these stakeholders was sought through public comments at a stage when the draft guidelines were fixed. However, for reflecting the stakeholder opinions in the main body of the guidelines, it would be better to incorporate them starting from the initial drafting stage. In this context, the ICH decided that the EWG would deeply involve external stakeholders in the process of ICH-GCP revision [6].

Stakeholder engagement was implemented in the United States, European Union, and Japan. The Ministry of Health, Labour and Welfare (MHLW) of Japan organized a special research group for reflecting the Japanese stakeholders’ opinions efficiently. The group conducted a survey on ICH-GCP among Japanese academic investigators and made recommendations for including the opinions of patients and the public. Here we summarize the survey results and recommendations.

## Methods

The special research group included 18 specialists on clinical trials, where at least 1 member joined from all of the 12 Clinical Research Core Hospitals as of March 2020. Clinical Research Core Hospitals are hospitals designated by MHLW to be the core of clinical trials in Japan.

During July 3 to August 7, 2020, a web-based survey was conducted among academic investigators and clinical trial support staff at 18 institutions, which included the Clinical Research Core Hospitals and the National Centers for Biomedical Research and Innovation, which are specialized centers of clinical trials in each disease field. The questions on the survey were shown in Table 1. The survey mainly focused on the necessity of revising the principles and sections of the current ICH-E6 (R2).

**Table 1:** Questions on the Survey

1. Respondent background Respondent’s name and contact information Institution, department Main job role (physician, clinical research coordinator, research ethics coordinator, biostatistician, project manager, monitor, or other) (Optional) Main research area 2. Principles of ICH Are any revisions needed for each principle in the current ICH-E6? Do you have any suggestions for adding new ICH principles? 3. Sections of ICH Are any revisions needed for each section in the current ICH-E6? Do you have any suggestions for adding new ICH sections? 4. General questions Do you have any expectations and concerns for the ICH-E6 revision? Are there any items in the current ICH-E6 considered to be disadvantageous for patients? How do you think patient involvement should help drug development? How should we solicit the opinions of patients and public on ICH-E6 revision? Did you experience any issues while conducting clinical trials during the COVID-19 pandemic? Do you have any other comments?	

Based on the results of the web-based survey, the special group members discussed whether the input from the survey results should be included in the recommendations. The special group made draft recommendations. The outline of the draft recommendations from the perspective of Japanese academics was presented and discussed at the EWG meeting on November 16, 2020.

On February 10, 2021, the special research group held a meeting with representatives of patients and the public, including major patient advocacy groups and members of the Research Ethics Committee of the National Cancer Center, to receive feedback on the draft recommendations. After incorporating their opinions, the recommendations were finalized and submitted to the EWG on March 23, 2021.

## Survey Results

A total of 105 academic stakeholders from 18 institutions responded to the survey. The participating institutions and respondents’ characteristics are summarized in Table 2.

**Table 2:** Participants' Profile

Sites	Number of respondents
Clinical Research Core Hospitals (13 sites)	
National Cancer Center Hospital East*	10
Juntendo University Hospital	9
Osaka University Hospital	7
Hokkaido University Hospital	6
Tohoku University Hospital	6
National Cancer Center Hospital*	6
Nagoya University Hospital	6
Okayama University Hospital	6
Kyushu University Hospital	6
Kyoto University Hospital	5
Chiba University Hospital	4
The University of Tokyo Hospital	4
Keio University Hospital	4
National Research Centers for Advanced and Specialized Medical Care (5 sites, excluding 2 duplicate sites*)	
National Center of Neurology and Psychiatry	7
National Center for Child Health and Development	7
National Cerebral and Cardiovascular Center	6
National Center for Geriatrics and Gerontology	4
National Center for Global Health and Medicine	2
Respondent’s main role in research	
Central support staff (project manager, monitor, pharmacovigilance, etc.)	32
Clinical research coordinator	20
Physician	19
Staff of institutional review board office	16
Biostatistician	13
Other (auditor, 2; secretariat, 2; ethics expert, 1)	5

*Two hospitals are designated as both Clinical Research Core Hospital and National Research Center for Advanced and Specialized Medical Care

**Fig. 1:**
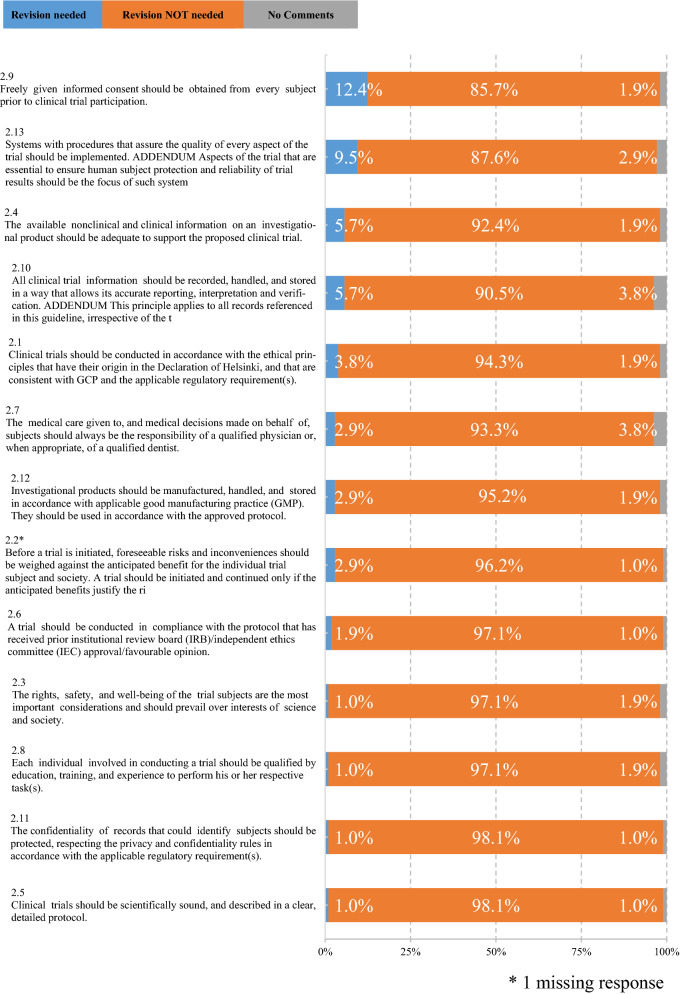
Respondents’ Recommendations for Revising the ICH E6 Principles (*n* = 105). *1 missing response

Figure 1 shows the necessity of revising the introduction and principles (Chapters 1–2) of the current ICH-E6 (R2). The top four principles identified as needing renovation were (i) informed consent (Chapter 2.9, 12.4% of respondents believed it needed renovation), (ii) systems for quality assurance (Chapter 2.13, 9.5%), (iii) information on an investigational product (Chapter 2.4, 5.7%), and procedures on clinical trial information (Chapter 2.10, 5.7%). Table 3 shows the comparison of the top five principles needing revision in the current Japanese survey and the similar survey conducted by the Clinical Trials Transformation Initiative (CTTI) [7], where the protection of the confidentiality of participant records and privacy was less focused in the Japanese survey. On the other hand, the top five principles that the respondents believed did not need renovation were (i) protocol (Chapter 2.5, 98.1% of the respondents believed it did not need revision); (ii) confidentiality (Chapter 2.11, 98.1%), (iii) qualification (Chapter 2.8, 97.1%), (iv) rights, safety, and well-being of the trial subjects (Chapter 2.3, 97.1%); (v) ethical review (Chapter 2.6, 97.1%).

**Table 3:** Top 5 Principles Needing Revision in CTTI Survey and the Japanese (JP) Survey

Top 5 principles in CTTI survey	Rank in JP survey	Top 5 principles in JP survey	Rank in CTTI survey
2.13 Implementing systems that assure quality	2	2.9 Obtaining informed consent	4
2.7 Providing medical care by a qualified physician or dentist	6	2.13 Implementing systems that assure quality	1
2.11 Protecting the confidentiality of participant records and privacy	12	2.4 Providing adequate the information on an investigational product	6
2.9 Obtaining informed consent	1	2.10 Documenting and storing clinical trial information to ensure accurate reporting, interpretation, and verification	5
2.10 Documenting and storing clinical trial information to ensure accurate reporting, interpretation, and verification	4	2.1 Conducting clinical trials in accordance with the ethical principles	11

**Fig. 2:**
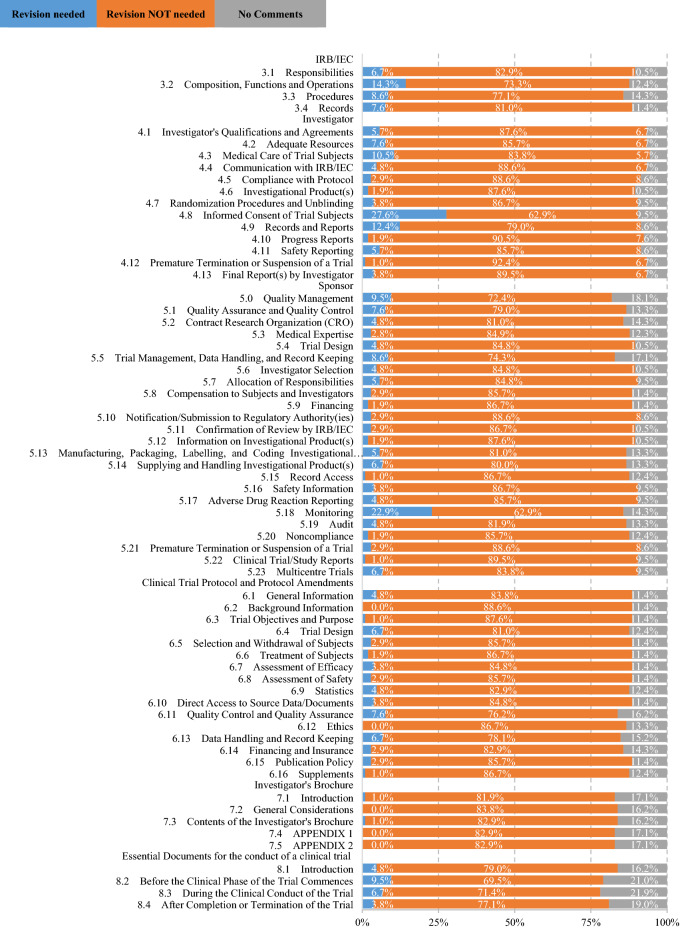
Respondents' Recommendations for Renovating the ICH E6 Sections (*n* = 105)

Figure 2 shows the necessity of revising procedural sections (Chapters 3–8) in the current ICH-E6 (R2). The top three sections identified as needing renovation were: (i) Investigator: informed consent (Chapter 4.8, 27.6% of respondents believed it needed renovation); (ii) Sponsor: monitoring (Chapter 5.18, 22.9%); (iii) Institutional Review Board (IRB)/Independent Ethics Committee (IEC): composition, functions, and operations (Chapter 3.2, 14.3%). Table 4 shows the comparison of the top five procedural sections needing revision in the current Japanese survey and the CTTI survey, where the essential documents during and after the trial period were less emphasized and the composition of IRB/IEC members aroused more interests in the Japanese survey. On the other hand, the top four sections that the respondents believed did not need renovation were: (i) Investigator: premature termination or suspension of a trial (Chapter 4.12, 92.4% of respondents believed it did not need renovation), (ii) Investigator: progress reports (Chapter 4.10, 90.5%), (iii) Sponsor: final report (Chapter 4.13, 89.5%), and (iv) Sponsor: clinical trial/study reports (Chapter 5.22, 89.5%).

**Table 4:** Top 5 Topics in the 66 Procedural Sections Needing Revision in CTTI Survey and the Current Japanese (JP) Survey

Top 5 principles in CTTI survey	Rank in JP survey	Top 5 principles in JP survey	Rank in CTTI survey
5.18 Sponsor: Monitoring	2	4.8 Investigator: Informed Consent of Trial Subjects	7
8.3 Essential Documents: During the Clinical Conduct of the Trial	14	5.18 Sponsor: Monitoring	1
8.4 Essential Documents: After Completion of Termination of the Trial	33	3.2 IRB/IEC*: Composition, Functions and Operations	16
5.5 Sponsor: Trial Management, Data Handling, and Record Keeping	8	4.9 Investigator: Records and Reports	6
8.2 Essential Documents: Before the Clinical Phase of the Trial Commences	6	4.3 Investigator: Medical Care of Trial Subjects	39

*IRB/IEC, Institutional Review Board/Independent Ethics Committee

## Recommendations on the Introduction and Principles Section

One of the most important recommendations was to clarify the scope of the ICH-GCP. The introduction of ICH-E6 (R2) stated that the purpose of the ICH-GCP guidelines is to promote mutual acceptance of clinical data by regulators in the European Union, Japan, and the United States. However, another part of the introduction stated that “this guideline may also be applied to other clinical investigations that may have an impact on the safety and well-being of human subjects.” These two statements make the scope of ICH-GCP ambiguous; it is unclear whether the scope of ICH-GCP is limited to data for the purpose of new drug applications (NDAs), or whether it extends to clinical trials in general. Since there are no other major standards for clinical trials that are used internationally, ICH-GCP is widely applied, even to clinical trials not intended for NDAs. This often leads to over-quality for clinical trials that are not necessarily mandated by regulatory agencies. Therefore, the scope of the ICH-GCP guidelines should be clarified, regarding whether it is limited to interventional studies of drugs aiming for NDAs, by deleting ambiguous statements such as “this guideline may also be applied to other clinical investigations.” Otherwise, if ICH-GCP intends to expand to other types of clinical trials, requirements for interventional studies of drugs for NDA purposes versus other studies should be specified separately. Even in the latter case, the compliance elements should be specified and proportionate to the purpose and risk of the study and the importance of the collected data.

The next recommendation is about proportionality, or fit-for-purpose flexibility in quality and procedures. For example, the principle of record storage stated in Chapter 2.10 states that “all” clinical trial information should be recorded, handled, and stored, and that this principle applies to “all” records referenced in this guideline, irrespective of the type of media used. In this regard, the research group recommended that the documentation to be recorded and retained should be narrowed down to “important” information, depending on the purpose and design of the study. Otherwise, the workload for record storage imposed on the study site will greatly exceed what is necessary to achieve the quality originally required. In the same manner, Chapter 2.13 states that systems with procedures that assure the quality of “every” aspect of the trial should be implemented. It is recommended that the scope should also be focused on assuring critical aspects of quality, depending on the purpose and design of the trial. Specifically, it would be better to state that procedures should be based on the quality required for the research.

## Recommendations on the Procedural Sections

In the current ICH-E6 (R2), Chapter 3.2 mentions composition, functions, and operation of the IRB/IEC. Here, the research group recommended maintaining the diversity and liquidity of IRB or IEC member composition. Diversity refers to, but is not limited to, diversity in gender, race, and culture. Liquidity means that IRB or IEC members should be replaced periodically. Although some countries or regions might have already implemented these practices, the statement will help emphasize the importance of diversity and liquidity in the composition of IRB or IEC members for the remaining countries or regions.

Regarding methods for modernizing informed consent from subjects (Chapter 4.8), the research group recommended emphasizing the importance of the informed consent process and improving the quality of consent, rather than focusing solely on the efficiency of obtaining informed consent for the sponsor and investigators. They also recommended facilitating the use of electronic consent, which involves providing explanations to subjects using electronic devices and obtaining electronic signatures. Since digital technologies could promote continuous communication between healthcare professionals and patients or subjects, and helping improve their satisfaction in clinical trials, the use of such digital technologies should be promoted.

Modernizing monitoring methods (Chapter 5.18), was also recommended. Conventional on-site monitoring performed is no longer the only standard method of monitoring; thus, various monitoring methods including central monitoring and remote monitoring should be incorporated. For example, Chapter 5.18.3 currently states that central monitoring may be determined by the sponsor in exceptional circumstances, but it is not necessary to limit it to exceptional circumstances. In the ICH-E6 (R2) addendum, a risk-based approach is incorporated; this idea should be incorporated more fully in E6 (R3). In terms of remote monitoring, various remote monitoring approaches have been tried recently. There is no consensus on the definition, procedures, and requirements of remote monitoring. Thus, when remote monitoring is incorporated in E6 (R3), clear definitions with descriptions of some example procedures are recommended.

Clinical trial registration was recommended as a new procedural chapter. From the perspective of publication bias, the study sponsor should register the required information in an accessible database prior to recruitment of the first subject. In addition, the information should be registered in the database in a timely manner so that patients or subjects and the public can easily access the necessary information when they want to know the enrollment status and results of clinical trials.

## Consideration on Annex 2 of ICH-E6 (R3)

The concept paper stated that interventional study using real-world data (RWD) as historical control data is expected to be incorporated in Annex 2 of ICH-E6 (R3). Thus, the research group recommended indicating how to set the required regulatory grade when RWD are used. Since RWD such as patient registries and electronic health records (EHRs) are not always designed for the purpose of regulatory submission, some examples of additional post hoc methods that can satisfy the regulatory grade might be illustrated. For example, adaptive monitoring might be useful for fulfilling the required regulatory grade, in which the target patients are extracted from RWD and additional monitoring is performed focusing on data items of interest. The same can be applied to pragmatic trials, which are also expected to be incorporated in Annex 2. Many pragmatic trials are initiated without any intention of regulatory submission, but sometimes regulatory usage is considered after the trial results are obtained. Therefore, it is preferable to specify minimum requirements for initiating these pragmatic trials and to indicate what requirements can be relaxed, for instance, monitoring and record storage.

## Discussion

The reported recommendations were developed based on a survey of more than 100 core Japanese academic stakeholders. The recommendations also reflected the opinions from representatives of patients and the public. The ICH reflection paper officially mentioned the necessity of stakeholder engagement for revising ICH-E6 [3]. Stakeholder engagement has been implemented in the United States, European Union, and Japan. In the United States, the CTTI conducted a comprehensive survey of stakeholders [7, 8]. The Food and Drug Administration held a public web conference with CTTI to collect stakeholder experiences [9]. In the European Union, the European Medicines Agency held a workshop to gather the views of patients, healthcare professionals, and clinical researchers [10]. As one of the founding members of ICH, the Japanese MHLW organized this special research group to conduct a survey on ICH-GCP with Japanese academic investigators, hold a public conference, and make recommendations reflecting the opinions from patients and the public.

The survey items were developed based on a similar survey conducted by CTTI. Specifically, the necessity of revising the principles and sections of ICH-E6 was specifically asked. According to the CTTI survey results, the top five principles needing renovation were (i) systems for quality assurance (Chapter 2.13), (ii) qualifications of physicians (Chapter 2.7), (iii) confidentiality of records (Chapter 2.11), (iv) informed consent (Chapter 2.9), and (v) procedures on clinical trial information (Chapter 2.10) [8]. The current Japanese survey results showed similar trends, but there were some differences as is shown in Tables 3 and 4. For example, only 1.0% (1/105) of respondents expressed concerns about the confidentiality of records, which ranked third in the CTTI survey. According to in-depth interviews by CTTI, regional and national variations in privacy rules and confidentiality in decentralized trials seemed to raise concerns [11], but it was not the case in Japan. Although there are some complicated privacy rules for observational studies in Japan, rules are standardized, at least in registration-directed trials. In addition, decentralized trials have not been common in Japan, which might be reasons for the differences. 

Informed consent was ranked as the top topic needing renovation in terms of principles and sections in Japan, whereas it was ranked fourth in terms of sections in most need of revision in the CTTI survey. Most concerns about informed consent raised by Japanese stakeholders were in the context of using RWD, because most of the medical information accumulated through daily practice is utilized for clinical research with opt-out consent methods. Thus, if RWD is directly to be used for regulatory purposes, the appropriateness of the opt-out methods will be debated. In this regard, the addition of Annex 3 was proposed in the 2017 ICH reflection paper with the aim of utilizing observational studies, patient registries, and alternative data sources [3]. However, Annex 3 was deleted from the concept paper in 2019, and the scope of Annex 2 was limited to interventional trials that incorporate RWD for reference [4]. Thus, concerns about informed consent from the Japanese stakeholders might reflect confusion on the scope of Annex 2. Nevertheless, many Japanese stakeholders expressed expectations of using RWD for regulatory purposes, and incorporation of stakeholder opinions is desired during the development of Annex 2.

Monitoring was ranked as second in terms of sections needing renovation in Japan, whereas it was the top section in the CTTI survey. Remote monitoring methods were particularly controversial. Various approaches to remote monitoring have been tried, especially during the COVID-19 pandemic. For example, only a few Japanese institutions have allowed direct access to EHRs; thus, sometimes a clinical research coordinator (CRC) copies the required data from the EHR and a monitor performs source data verification using the copied data. In another example, a CRC displays EHRs and communicates with a monitor through an online video conferencing system. Remote monitoring procedures have not yet been well standardized, but remote monitoring will certainly become more important for improving the efficiency of clinical trials in the future. Thus, there should be a global consensus about standard methods and considerations for remote monitoring.

There are three major regulations on clinical trials in Japan: (i) the Japanese GCP based on the Pharmaceutical and Medical Devices Act, (ii) the Clinical Trials Act, and (iii) the Ethical Guidelines for Medical and Biological Research Involving Human Subjects [12]. The Japanese GCP will be amended based on the GCP renovation. The Clinical Trials Act and the Ethical Guidelines would also be influenced indirectly. Although there are a few exceptions, only clinical trial data from registration-directed trials under the Japanese GCP are basically accepted by the Pharmaceuticals and Medical Devices Agency, the Japanese regulatory authority. Through the GCP renovation, all drug interventional studies will be incorporated into the scope of ICH-GCP. In addition to the recent evolution of Japanese regulatory frameworks [13], even clinical trial data under the Clinical Trials Act will likely be used for regulatory purposes. Since most clinical trials under the Clinical Trials Act are not primarily intended for regulatory submission, excessive quality should be avoided. However, what regulatory grade is required and whether there are post hoc procedures that can fulfill the required data quality grade are questions that should be discussed.

## Conclusion

In these recommendations for GCP renotation, we proposed the necessity of clarification of the scope of ICH-GCP, proportionality in various aspects of clinical trials, diversity and liquidity of ethics committee members, modernization of informed consent procedures, variations in monitoring, and regulatory grade in using real world data. Since academic stakeholders and patients are essentially involved in a clinical trial, the opinions from such stakeholders will strengthen the robustness of the GCP renovation.
